# Evidence for a Peak Shift in a Humoral Response to Helminths: Age Profiles of IgE in the Shuar of Ecuador, the Tsimane of Bolivia, and the U.S. NHANES

**DOI:** 10.1371/journal.pntd.0001218

**Published:** 2011-06-28

**Authors:** Aaron D. Blackwell, Michael D. Gurven, Lawrence S. Sugiyama, Felicia C. Madimenos, Melissa A. Liebert, Melanie A. Martin, Hillard S. Kaplan, J. Josh Snodgrass

**Affiliations:** 1 Integrative Anthropological Sciences, University of California, Santa Barbara, Santa Barbara, California, United States of America; 2 Department of Anthropology, University of Oregon, Eugene, Oregon, United States of America; 3 Institute of Cognitive and Decision Sciences, University of Oregon, Eugene, Oregon, United States of America; 4 Department of Anthropology, University of New Mexico, Albuquerque, New Mexico, United States of America; Leiden University Medical Center, Netherlands

## Abstract

**Background:**

The peak shift model predicts that the age-profile of a pathogen's prevalence depends upon its transmission rate, peaking earlier in populations with higher transmission and declining as partial immunity is acquired. Helminth infections are associated with increased immunoglobulin E (IgE), which may convey partial immunity and influence the peak shift. Although studies have noted peak shifts in helminths, corresponding peak shifts in total IgE have not been investigated, nor has the age-patterning been carefully examined across populations. We test for differences in the age-patterning of IgE between two South American forager-horticulturalist populations and the United States: the Tsimane of Bolivia (n = 832), the Shuar of Ecuador (n = 289), and the U.S. NHANES (n = 8,336). We then examine the relationship between total IgE and helminth prevalences in the Tsimane.

**Methodology/Principal Findings:**

Total IgE levels were assessed in serum and dried blood spots and age-patterns examined with non-linear regression models. Tsimane had the highest IgE (geometric mean  = 8,182 IU/ml), followed by Shuar (1,252 IU/ml), and NHANES (52 IU/ml). Consistent with predictions, higher population IgE was associated with steeper increases at early ages and earlier peaks: Tsimane IgE peaked at 7 years, Shuar at 10 years, and NHANES at 17 years. For Tsimane, the age-pattern was compared with fecal helminth prevalences. Overall, 57% had detectable eggs or larva, with hookworm (45.4%) and *Ascaris lumbricoides* (19.9%) the most prevalent. The peak in total IgE occurred around the peak in *A. lumbricoides*, which was associated with higher IgE in children <10, but with lower IgE in adolescents.

**Conclusions:**

The age-patterning suggests a peak shift in total IgE similar to that seen in helminth infections, particularly *A. lumbricoides*. This age-patterning may have implications for understanding the effects of helminths on other health outcomes, such as allergy, growth, and response to childhood vaccination.

## Author Summary

Infection with parasitic worms, known as helminths, alters the immune system, causing individuals to produce high levels of a type of antibody known as immunoglobulin E (IgE). IgE is typically very low in western populations, but is many times higher where helminth infections are common, particularly indigenous populations in South America. Helminths infect more than one seventh of the world's population. Since helminths tend to infect people at younger ages in areas where they are more common, a disproportionate number of those affected are schoolchildren. In this paper we examine IgE levels in two indigenous South American groups in comparison to levels in the United States. In these groups we find that IgE levels are not only higher, but that they also reach their highest levels at earlier ages in more infected populations. This finding is important since effects on immune function, including IgE production, may have additional consequences if they occur at young ages, changing the development of allergy and asthma, growth, and response to vaccines.

## Introduction

Age-related epidemiological patterns are thought to result from complex interactions between parasite life cycle, exposure to infection, and host-immunity [1], [2]. Helminth infections show characteristic age-patterning, peaking around puberty and then declining during adulthood [3]–[5]. However, this pattern varies with infection prevalence and intensity, tending to both peak and decline earlier in populations with higher rates of transmission. This “peak shift” is thought to result from the interaction between the rate at which new individuals are infected and the rate at which partial immunity is acquired [1], [2]. According to this model, when transmission is high infection occurs more quickly, leading to a higher prevalence at a younger age. However, earlier infection also leads to an earlier acquisition of immunity, leading to a decline in prevalence following the peak.

Although studies have found age-patterns in helminth prevalences consistent with this hypothesis, few studies have examined whether the age-patterning of immune responses follows similar patterns. Those studies that have examined the age-patterning of immune responses have generally focused on parasite specific immunoglobulins (IgG, IgA, IgM, and IgE) [6]–[11]. These have shown age-patterns that resemble the age-specific prevalences of parasites. However, in addition to specific responses, helminth infections are associated with a general shift in the host immune system towards a T_H_2-biased phenotype, characterized in particular by increased production of total IgE. Although specific responses are thought to participate in protection against infection and thus the generation of peak-shift patterns, an examination of total IgE levels is also critical for understanding the effects of helminths on health and immune function. Due to stimulation of T_H_2 responses, total IgE is likely to represent the total burden of multiple helminth species infection better than species specific immunoglobulins. Like specific IgE, total IgE levels are elevated in infected individuals and fall with treatment [12], [13], and have been shown to correlate with specific IgE for *Ascaris lumbricoides* and *T. trichiura* [14]. However, total IgE levels in heavily parasitized individuals remain elevated compared to individuals in industrialized countries for substantial periods of time [15], suggesting persistent changes in host immune function.

Mounting an immune response is energetically costly, necessitating reductions in competing life history demands, including growth, reproduction, and survival [16]–[17]. Higher total IgE levels are associated with poorer growth and shorter adult stature, suggesting a trade-off between growth and investment into immune response [18]. Moreover, the shifting of immune function towards a T_H_2 phenotype may reduce T_H_1 responses, decreasing the effectiveness of vaccines or increasing susceptibility to viruses and bacteria [19]–[21].

These effects may depend, in part, on the timing of exposure, as exposure to helminths during critical periods may bias the development of immune function or a child's growth trajectory. Helminths infect more than one seventh of the world's population, and given the peak-shift pattern, a disproportionate number of those infected are schoolchildren [22]. As a consequence, age-patterns in helminth infection and immune response are likely to have significant consequences on growth and development.

Although several studies have reported that IgE increases quickly in the first 5–10 years of life and then levels off [23], [24], few studies have carefully examined the age-patterning of total IgE and we know of no published studies that have compared age-patterning in IgE across multiple populations. As a marker of helminth infection and T_H_2-biasing of T-cell responses, an understanding of the age-patterning of total IgE is important for understanding the broader consequences of helminth infections on life history parameters. The current study describes in detail the age-patterning of IgE levels in three populations. These include data from the United States collected by the National Health and Nutrition Examination Survey 2005–2006 (NHANES) and data from two populations of South American forager-horticulturalists: the Tsimane of Boliva and the Shuar of Ecuador. First, we test for predicted associations between population mean IgE level and the age-pattern of IgE. Second, using Tsimane data we examine the relationship between age-patterning in IgE and age-patterning in helminth infections.

## Methods

### Study Populations

#### Shuar

Shuar are Amerindians from the Amazonas region of Ecuador [25], [26]. Shuar live across a wide range of circumstances, but a large portion of the population continues traditional subsistence based on horticulture, hunting, and fishing. Approximately 40% of Shuar children are stunted, a higher prevalence than is found in other indigenous and non-indigenous children living in the same area [27]. Although we know of no studies examining helminth infections in the Shuar, recent studies report infection rates of around 50% in other Amazonian Ecuadorian populations, with *Ascaris* the most prevalent parasite [28], [29]. Shuar data were collected as part of the Shuar Life History Project (www.bonesandbehavior.org/shuar) in a village that has been previously described [18].

#### Tsimane

Tsimane are forager-horticulturalists that live along the Maniqui River in lowland Bolivia. Tsimane subsist primarily on cultivation of plantains, rice, manioc, and corn, as well as hunting, fishing, and gathering. Tsimane show high levels of inflammatory markers, such as C-reactive protein [30]–[32]. Helminth infections are highly prevalent, with hookworm (*Necator americanus* or *Ancylostoma duodenale*) being the predominant parasite, infecting between 44% and 76% of children [33], [34]. Between 40–50% of children are stunted [35], [36]. The data for this study were collected as part of the Tsimane Health and Life History Project (http://www.unm.edu/~tsimane/), in sixteen villages representing a range of environmental and economic situations (interior forest, riverine, acculturated, non-acculturated).

#### National Health and Nutrition Examination Survey (NHANES)

NHANES is a large-scale, national survey of health, nutrition, and social factors conducted by the National Center for Health Statistics and Center for Disease Control. This study uses data from the NHANES 2005–2006 dataset (http://www.cdc.gov/nchs/nhanes/nhanes2005-2006/nhanes05_06.htm). The sample includes 8,336 individuals, 88% percent U.S. citizens, 52% percent female, 27% Mexican-American, 36% Caucasian, 26% African-American, and 11% other ethnicities.

### Ethics Statement

For Shuar, permission to conduct the study was first obtained from the Federacíon Interprovincial de Centros Shuar (FICSH), the elected representational organization for Shuar affairs. Second, permission was obtained from elected village leaders. Third, a village meeting was held in which a village-level consent form was read aloud, the study explained, questions answered, and a community decision reached about whether to allow the study. Individuals were informed that they could choose not to participate, participate only in individual portions of the study, or participate in the full study. At the time of data collection, individual oral consent was obtained, with individuals able to opt-in or out of individual components of the study (e.g., to provide blood spots or not). For subjects under age fifteen (the local age of consent) both parental consent and child assent were obtained. Oral consent was used for two reasons: 1) many Shuar are non or semi-literate or have only a few years of schooling, and 2) many Shuar are suspicious or uncomfortable with signing documents due to a history of territorial land disputes and wariness about signed documents leading to ownership conflicts. An independent bilingual Shuar village leader, nurse, FISCH official or assistant was present to translate as needed during group and individual consent and study procedures. The study and consent procedures were approved by the Institutional Review Board (IRB) of the University of Oregon.

For Tsimane, informed consent was obtained at three levels: 1) from the Gran Consejo Tsimane, the local Tsimane government organization that represents Tsimane interests and oversees all projects, 2) community officials and participants in village meetings, and 3) individual consent during medical visits and before each procedure. After explanation of a formal protocol by bilingual Tsimane assistants, consent forms were signed for literate participants, and verbal approval with fingerprint signature given for non-literate participants. Tsimane consent procedures were approved by the IRBs at the University of New Mexico, University of California, Santa Barbara and the University of Southern California.

### Blood Collection and Analysis

#### Shuar

Shuar samples were collected following standard procedures to collect dried blood spots [37]. IgE levels were determined by ELISA at the University of Oregon, following a commercially available protocol (Bethyl Labs, Inc.: #E80-108 and #E101) adapted for use with blood spots [38]. Blood spot collection and IgE analyses have been previously described [18].

#### Tsimane

Tsimane blood samples were collected by venipuncture during annual medical exams conducted by Bolivian physicians. Serum was frozen in liquid nitrogen for transport to New Mexico. Two rounds of samples were collected. The first 223 samples were collected in 2004–2005 and analyzed by TriCore Laboratories (Albuquerque, NM) for total IgE (catalog: L2KIE6) using an Immulite 2000 (Siemens Corp; Deerfield, IL). An additional 700 samples were collected in 2007 and analyzed in the laboratory of JJS at the University of Oregon using the same commercial ELISA kit used for Shuar samples (Bethyl Labs, Inc.: #E80-108 and #E101). Of these, 91 were repeated measures for individuals included in the first batch of samples. These samples were excluded so as not to confound longitudinal and cross-sectional data. After excluding these cases, the first and second samples did not differ in geometric mean IgE (comparison of log transformed IgE: t = .462, df = 830, p = .644).

#### NHANES

NHANES samples were collected by venipucture by trained phlebotomists. Determination of total IgE was done using the ImmunoCAP 1000 system (Pharmacia Diagnostics) by the Department of Pathology Immunology Laboratory at Elmhurst Memorial Hospital, Elmhurst, IL. Details can be found at http://www.cdc.gov/nchs/data/nhanes/nhanes_05_06/al_ige_d_met_specific_ige_total_ige.pdf.

#### Comparability of Blood Samples

A handful of studies have compared IgE in dried blood spots to IgE measured in serum and found results to be virtually identical [39], [40]. Additionally, the ELISA procedure used to determine blood spot IgE in this study has been validated against controls with known IgE levels [38]. To verify the comparability of IgE obtained from dried blood spots with that obtained from serum, six matched blood spot and serum samples were analyzed using both methods. The values obtained from dried blood spots were highly correlated with the values from serum (r = 0.98, p<.001). Blood spot values were ∼3% higher. Using linear regression, the following conversion factor was obtained to convert dried blood spot values into serum values prior to other analyses: IgE_serum_ = 0.965×IgE_DBS_−3.458 (IU/ml).

### Fecal Analysis

Tsimane fecal samples were analyzed using two methods. From 2004 to 2008 fecal samples were analyzed for the presence of helminth eggs and larvae by direct identification on wet mounts. As described in greater detail elsewhere [33], duplicate mounts were prepared with 0.9% saline solution and iodine solution, respectively, and examined at 100x and 400x for helminth eggs (hookworm, *A. lumbricoides,* and *T. trichiuris*), and larvae (*S. stercoralis*). Beginning in 2007, fecal samples were also preserved in 10% formalin solution following direct identification, and later quantitatively analyzed using a modified Percoll (Amersham Pharmacia) technique [41].

Of the two methods, the Percoll technique is more sensitive, producing slightly higher detection rates than direct identification (59.4% vs. 51.9% infected). These differences may be due to the greater efficiency of the Percoll technique in detecting eggs in fibrous stools and at low-intensities [41]. However, for the present study the differences between the two methods were not qualitatively great enough to justify using only data produced by one method or the other. We therefore aggregated data from the two methods, coding individuals as either infected or not infected if helminths were detected by either method. In total 1,495 individuals had Percoll results, with the remaining 3,610 having only direct results.

### Age Estimation

Birth dates accurate to the month were available from health clinic and school records for most Shuar children. For Shuar adults, birth dates on government identification were cross-checked with extensive genealogical information collected from multiple informants. Tsimane genealogies were collected during demographic interviews done on individuals over age 18 (n = 1,098). Tsimane ages were estimated based on written records, such as those kept by Catholic missionaries, demographic interviews with independent cross-checking of genealogies and reproductive histories with multiple informants, and the use of photographs of people with known ages [42].

### Data Analysis

**Figure 1:**
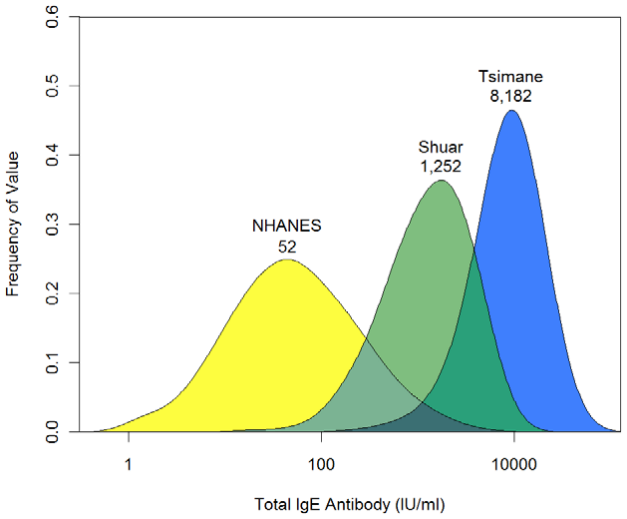
Distribution and geometric mean value of IgE antibody levels in three populations. Density plots were generated with a Gaussian smooth with bandwidth 0.5.

Prior to data analysis, IgE values were converted into international units (1 IU = 2.4 ng/ml). IgE is log-normally distributed in all three populations (Figure 1), so values were natural log transformed (lnIgE) before all analyses. For t-tests, reported means are geometric means calculated by taking the exponential of the mean log values used in the t-test. Descriptive statistics and t-tests were done in PASW Statistics 18.0 (formerly SPSS Statistics, SPSS Inc.). All other analyses were done in R 2.10.1 (www.r-project.org).

**Figure 2:**
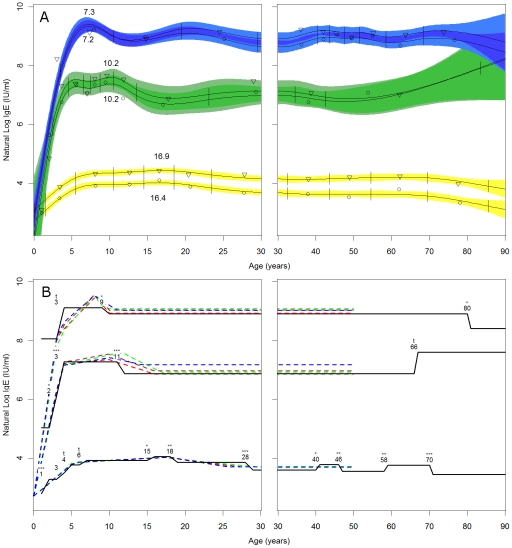
Models for IgE by age in Tsimane, Shuar, and NHANES. A) Generalized additive models for Tsimane (top, blue), Shuar (middle, green), and NHANES (bottom, yellow). Points show the mean lnIgE value for males (triangles) and females (circles) between knots specified in the initial model basis (vertical lines), while lines indicate the thin plate regression spline for each sex. For all three populations males have the higher fit line. Numbers indicate the estimated ages at which the initial peak in IgE occurs. Shading indicates local 95% confidence intervals for the spline, with dark areas indicating overlap between male and female confidence intervals and light areas indicating no overlap. B) Ordinal step models and non-linear regression models. Ordinal model parameters were entered in stepwise fashion according to AIC minimization, resulting in the final models. Numbers indicate a significant transition at greater than the age given, symbols the significance of the parameter in the model: ^t^ p≤0.10, * p≤0.05, ** p≤0.01, *** p≤0.001. Dashed lines indicate the model fits for non-linear models, including the population specific models in Table 3 (red), and the interaction models in Table 4 (Model 1 in green, Model 2 in brown, and Model 3 in blue). For simplicity only the models for females are shown.

Generalized additive models (GAM; [43], [44]) were used to examine the non-parametric age pattern of IgE levels for each population. Models were fit with the *gam* procedure in package *mgcv* using thin plate regression splines [45], [46]. Since the cases in each population were not evenly distributed by age, initial basis knots were specified for each population based on even ten-percentiles of the age distribution, allowing knots to be spaced with an equal number of cases between them (Figure 2). Apart from the basis knots, smoothing parameters were generated automatically according to *gam* defaults [45]. GAM models included an intercept, a sex factor, a spline for age, and a spline for age-by-sex interaction.

In initial models, similar IgE levels at birth were predicted among Shuar and NHANES, with the Shuar model predicting IgE of 7 IU/ml for females and 9 IU/ml for males, and the NHANES model predicting 15 IU/ml for females and 21 IU/ml for males. However, due to the relatively low number of Tsimane under age five, initial Tsimane models were essentially straight lines, with peak IgE predicted at birth. A number of studies have found extremely low IgE levels at birth (<1 IU/ml) [47]-[50], [23], [51], [52], even in infants of mothers with helminth infections and high IgE [53], [54]. Given the convergence of the other two models and these previous findings, we used dummy cases with age zero and IgE equal to 15 IU/ml to anchor Tsimane models to a similar intercept at birth. Dummy cases were included in GAM models but not in any other statistic.

GAM models with a binomial logit-link function were also used to estimate odds-ratios for Tsimane helminth infection by age. Associations between helminth infection and IgE levels were estimated in linear models controlling for infection with other helminths, sex, and age.

In addition to GAM, two other methods were used to verify age shapes and compare populations. In the first, a stepwise linear regression was used to identify critical age-related changes in lnIgE for each population. Dummy variables were coded for each unique age indicating whether a case was greater than the given age (e.g., [55]). Starting from a model with only an intercept and sex term, *stepAIC* (package MASS) was used to enter and remove age variables to minimize model AIC [56].

For the second test we constructed non-linear models composed of linear segments linked together, with model terms representing the point at which the linear segments are stitched together. In this model, model terms directly represent critical ages, such as the age at which the model peaks, so differences in critical ages between populations can be tested using population interaction terms. The basic model is:



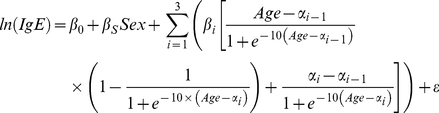
Where α_1_, α_2_ and α_3_ are the ages at which the slope changes and α_0_ equals zero, β_1_, β_2_, and β_3_ are the slopes for the segments, and additional terms β_0_ and β_s_ represent the intercept at age zero and sex effect, respectively. Three logistic functions serve to “turn-on”, “turn-off”, and maintain the value reached at each age transition. The models were fit such that α_1_ is the initial age where rapid increases in IgE level out, α_2_ is the age at which IgE peaks for the population, and α_3_ is the age at which IgE reaches mean adult levels. Models were solved using *nls* (package stats) using the nl2sol algorithm. Initial values specified based on GAM regressions and only individuals under age 50 were used for modeling.

## Results

### Age-Pattern of IgE

Of the three populations, Tsimane had the highest IgE levels (geometric mean  = 8,182 IU/ml), followed by Shuar (1,252 IU/ml), and NHANES (52 IU/ml) (Table 1). IgE distributions were skewed but largely normalized by log-transformation (Figure 1). All three groups differed from one another in pair-wise comparisons (all t-test p-values <.001 after Bonferroni correction). In all three populations, males had higher IgE than females. NHANES males had IgE levels 60% higher than females (65.9 vs. 41.3 IU/ml, t = 14.08, p<.01), while Shuar males had IgE values 29% higher than Shuar females (1,457 vs. 1,129 IU/ml, t = 2.15, p = .03), and Tsimane males had levels 16% above females (8,720 vs. 7,527 IU/ml, t = 2.73, p<.01).

**Table 1:** Sample sizes and IgE by population and age category.

	Tsimane			Shuar			NHANES		
Ages	N	Geometric Mean		N	Geometric Mean		N	Geometric Mean	
1–5	11	7,073	(1,078–46,426)	50	1,017	(127–8,124)	938	34	(2–706)
6–10	79	9,755	(1,989–47,847)	102	1,658	(308–8,920)	774	61	(3–1,288)
11–15	51	7,798	(1,788–34,008)	35	1,307	(172–9,927)	1,110	64	(3–1,380)
16–20	38	7,820	(2,411–25,364)	12	1,055	(109–10,228)	1,110	69	(3–1,453)
21–30	82	8,068	(1,641–39,670)	22	882	(64–12,205)	931	53	(2–1,131)
31–40	90	7,887	(1,594–39,031)	36	1,074	(178–6,491)	778	47	(3–882)
41–50	219	8,321	(1,797–38,534)	17	1,024	(167–6,289)	763	50	(2–1,058)
51–60	119	7,861	(1,385–44,612)	5	445	(78–2,547)	598	49	(3–763)
61–70	82	8,018	(1,534–41,916)	5	1,738	(182–16,623)	615	54	(2–1,319)
71–80	48	9,037	(2,590–31,537)	3	2,327	(651–8,311)	427	39	(2–863)
81–90	12	5,068	(620–41,428)	2	1,767	(160–19,485)	292	41	(2–1,028)
**Total**	**831**	**8,182**	**(1,691–39,582)**	**289**	**1,252**	**(172–9,118)**	**8,336**	**52**	**(2–1,116)**

Geometric mean values are in IU/ml. Values in parenthesis are plus or minus two standard deviations for log transformed values: e^µ±2*σ^, where µ is the mean of ln(IgE) and σ is the standard deviation of ln(IgE).

Upon initial visual examination of the data, age patterns were observed to be non-linear. We therefore used thin plate regression splines in GAM models to examine the age patterning of IgE (Figure 2A). Age terms were significant in all models (Tsimane: edf = 10.94, F = 43.15, p<.001; Shuar: edf = 7.73, F = 5.35, p<.001; NHANES: edf = 8.93, F = 24.74, p<.001). Despite differences in level, all three populations had similar age-related IgE profiles, characterized by a rapid increase before age five, a peak in the juvenile or adolescent period, and a decrease into adulthood. However, a number of features differ between populations. Principal among these is the age at which IgE initially peaks. Tsimane IgE peaked at 7.3 years for males and 7.2 years for females. Shuar IgE peaked at 10.2 for both sexes. NHANES IgE did not peak until age 16.9 for males and 16.4 for females. Fitting a linear model to the three population points for each sex suggested that for males the peak age decreases by 1.98 years for every one unit increase in population mean lnIgE (t = 18.40, p = 0.04), while for females the peak age decreases by 1.76 years per unit increase in lnIgE (t = 25.25, p = 0.03).

We next used stepwise linear regression with ordinal age variables to identify ages at which important transitions in IgE level occur and to test the significance of these changes (Figure 2B). Tsimane transitions included an increase at age three (β = 1.06, t = 1.90, p = 0.06) followed by a decrease after age nine (β = −0.21, t = −2.26, p = 0.02). For Shuar, there were significant increases after age two (β = 1.07, t = 2.10, p = 0.04) and age three (β = 1.18, t = 3.75, p<0.01), and a significant decrease after age eleven (β = −0.41, t = −3.59, p<0.01). In the NHANES sample increases were present in the model after age one (β = 0.48, t = 3.82, p<0.01), age three (β = 0.22, t = 1.60, p = 0.10), age four (β = 0.27, t  =  1.90, p  =  0.05), age six (β  =  0.15, t  = 1.67, p  =  0.10), and age fifteen (β = 0.13, t = 2.00, p = 0.04), with a decrease after age eighteen (β = −0.19, t = −2.79, p<0.01).

Since neither of these models directly tests for differences between populations or allows for the comparison of shape differences in age curves or peaks, we devised a non-linear modeling procedure in which four linear segments are used to model the age profile (Figure 2B). These models include three ages points that correspond to the point at which the rapid increases in early life levels off (α_1_), the age at which IgE peaks in the population (α_2_), and the age at which IgE reaches adult levels after the peak (α_3_). Three slopes (β_1–3_) describe the change in IgE between age points (birth – α_1_, α_1_ to α_2_, and α_2_ to α_3_). A sex term accounts for the difference between males and females (β_s_).

**Figure 3:**
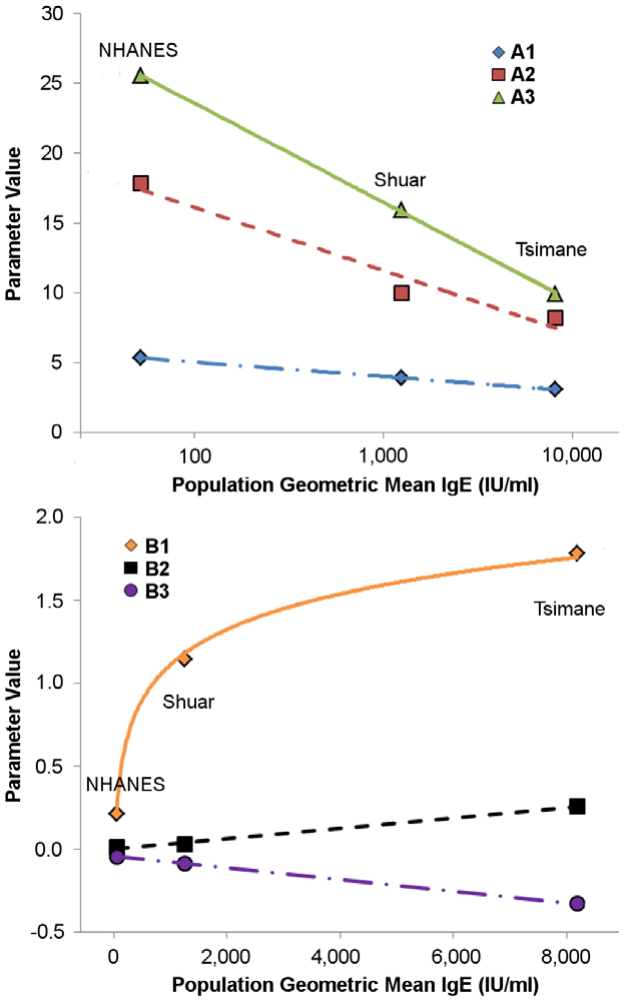
Association between model parameters and population geometric mean IgE levels. The upper panel shows fits between age parameters and mean log IgE by population. The lower shows fits between slope parameters and geometric mean IgE by population. Note that the fit for B1 is linear with regard to log IgE, but is shown on the lower graph due to the parameter scale. Correlation coefficients for all parameters are given in the text.

Models were first fit for the three populations independently (Table 2). Model parameters conformed well to predictions from GAM models, with peak ages (α_2_) of 8.2, 10.0, and 17.9 predicted for Tsimane, Shuar, and NHANES respectively. The ages of initial slope change and final adult level also corresponded to peak ages, with both ages earliest in Tsimane and latest in NHANES. Moreover, all model parameters were highly correlated with IgE levels (Figure 3). Age terms, initial slopes from age zero, and sex differences all correlated with mean log IgE (α_1_: r = −1.00,p<0.01; α_2_: r = −0.98, p = 0.13; α_3_: r = −1.00,p = 0.01; β_1_: r = 0.99, p = 0.02; β_s_: r = −0.99, p = 0.09), while the increase between the first peak and the final peak, and the decrease from the final peak to adult levels correlated with untransformed population geometric mean IgE (β_2_: 1.00, p = 0.06, β_3_: −1.00, p<0.01).

**Table 2:** Non-linear model parameters by population.

Population	Parameter	Estimate	SE	t-value	p
NHANES	β_1_ (Initial Slope)	.21	.02	13.57	<.001
	β_2_ (Second Slope)	1.43×10^−2^	9.09×10^−3^	1.58	.115
	β_3_ (Post-peak Decline)	−4.24×10^−2^	1.83×10^−2^	−2.32	.021
	α_1_ (Age of slope change)	5.33	.52	10.21	<.001
	α_2_ (Age of peak)	17.85	1.56	11.41	<.001
	α_3_ (Age adult level reached)	25.55	2.52	10.13	<.001
	β_S_ (Male vs. Female)	.44	.04	11.95	<.001
Shuar	β_1_ (Initial Slope)	1.15	.09	12.62	<.001
	β_2_ (Second Slope)	2.73×10^−2^	5.04×10^−2^	.54	.588
	β_3_ (Post-peak Decline)	−8.25×10^−2^	8.64×10^−2^	−.96	.340
	α_1_ (Age of slope change)	3.91	.36	10.94	<.001
	α_2_ (Age of peak)	9.99	3.13	3.19	.002
	α_3_ (Age adult level reached)	15.94	4.29	3.72	<.001
	β_S_ (Male vs. Female)	.22	.11	2.02	.044
Tsimane	β_1_ (Initial Slope)	1.78	.23	7.86	<.001
	β_2_ (Second Slope)	.26	.09	2.86	.004
	β_3_ (Post-peak Decline)	−.33	2.20	−.15	.882
	α_1_ (Age of slope change)	3.06	.53	5.82	<.001
	α_2_ (Age of peak)	8.21	2.85	2.88	.004
	α_3_ (Age adult level reached)	9.92	6.18	1.61	.109
	β_S_ (Male vs. Female)	.15	.06	2.30	.022

To compare populations on these terms we first attempted to fit models with population interaction terms for each parameter. However, this model, with 21 parameters, was too complex for the model algorithms and the data available, and failed to fit. Instead we simplified the models based on the relationship between model parameters and population mean IgE levels. In the first of these models we included parameter by population IgE interaction terms (Table 3, Model 1). This model verified interactions between population IgE and all model parameters, with each one unit increase in log IgE associated with a 0.37 year decrease in the age of the initial slope change, a 1.70 year decrease in the age of peak IgE, and a 4.10 year decrease in the age at which levels dropped to adult mean values. The initial rate of increase in IgE was also significantly related to IgE mean levels indicating bother faster and earlier acquisition of high IgE in the Tsimane and secondarily the Shuar.

**Table 3:** Four models comparing age profile parameters among NHANES, Shuar, and Tsimane.

		Model 1		Model 2		Model 3	
Parameter	Unit	Estimate	SE	Estimate	SE	Estimate	SE
β_1_-intercept	10^−1^ x IU/ml/yr	−9.22***	1.16	−9.82***	1.50	−10.13***	0.96
β_1_-slope	10^−1^ x IU/ml/yr/ ln(IgE)	2.89***	0.30	3.03***	0.37	3.11***	0.24
β_2_-intercept	10^−2^ x IU/ml/yr	1.44	0.93	1.26	0.88	1.29^t^	0.79
β_2_-slope	10^−5^ x IU/ml/yr/Mean IgE	3.50^t^	1.83	2.96	2.08	2.52	2.05
β_3_-intercept	10^−2^ x IU/ml/yr	−2.19**	0.82	−3.01*	1.48	−4.08***	0.78
β_3_-slope	10^−5^ x IU/ml/yr/ Mean IgE	−8.47^t^	4.80	−5.20	10.33	−1.44	1.15
α_1_-intercept	Years	6.55***	.92				
α_1_-slope	Years/Mean ln(IgE)	−0.37*	.15				
α_2_-intercept	Years	23.70***	3.48				
α_2_-slope	Years/Mean ln(IgE)	−1.70***	.39				
α_3_-intercept	Years	46.12***	5.94				
α_3_-slope	Year/Mean ln(IgE)	−4.10***	.66				
β_S_ (Male)	10^−1^ x IU/ml	6.77***	1.09	6.84***	1.10	6.77***	1.10
β_S-slope_	10^−1^ x IU/ml/Mean ln(IgE)	−0.59*	.23	−0.61**	.24	−0.59*	.23
α_1_ (Shuar)	Years			3.85***	.42		
α_1_ (NHANES vs. Shuar)	Years			1.49*	.64		
α_1_ (Tsimane vs. Shuar)	Years			−0.64^t^	.35		
α_2_ (Shuar)	Years			9.85***	2.34	10.09***	.94
α_2_ (NHANES vs. Shuar)	Years			7.66**	2.89	7.66***	1.27
α_2_ (Tsimane vs. Shuar)	Years			−1.47	1.98	−2.08*	1.04
α_3_ (Shuar)	Years			15.92**	5.96		
α_3_ (NHANES vs. Shuar)	Years			11.63^t^	6.65		
α_3_ (Tsimane vs. Shuar)	Years			−6.21	4.89		

All models are non-linear regression models of the form given in the Methods section of this paper. In Model 1 all parameters were entered as functions of population IgE level. For Models 2 and 3 age parameters were instead entered with population identity interaction terms. In Model 3 α_1_ and α_3_ were entered as functions of α_2_. Two-sided t-test significant levels:

^t^p≤0.10.

*p≤0.05.

**p≤0.01.

***p≤0.001.

In the second, third, and forth models we tested population differences in the ages at which slopes change, using population factor terms rather than interactions with population IgE. In Model 2 all three ages were left independent and the Shuar were used as a contrast group, since they lie between Tsimane and NHANES. In this model NHANES α_1_ and α_2_ were significantly later than Shuar ages, and α_3_ was later but with marginal significance. Although all three Tsimane ages were early than Shuar ages, none were significantly so, although all three were significantly earlier than NHANES ages when the model was run with NHANES as the contrast group (not-shown). Given the strong correlation between all three ages and mean IgE levels, we suspected that multicollinearity between terms might be reducing parameter significance. We therefore examined how α_1_, α_2_, and α_3_ might be included as functions of a single age term. By fitting linear models to the parameters in Table 3 we found that α_1_∼1.47+α_2_×0.22, and α_3_∼0.90+α_2_×1.50. We used these terms in Model 3, removing α_1_ and α_3_. In this model with a single age term to describe the shape, ages in both Tsimane and NHANES were significantly different from Shuar ages, with the overall age shape shifted earlier in Tsimane and later in NHANES.

### Tsimane Age-Pattern of Helminth Infection

**Figure 4:**
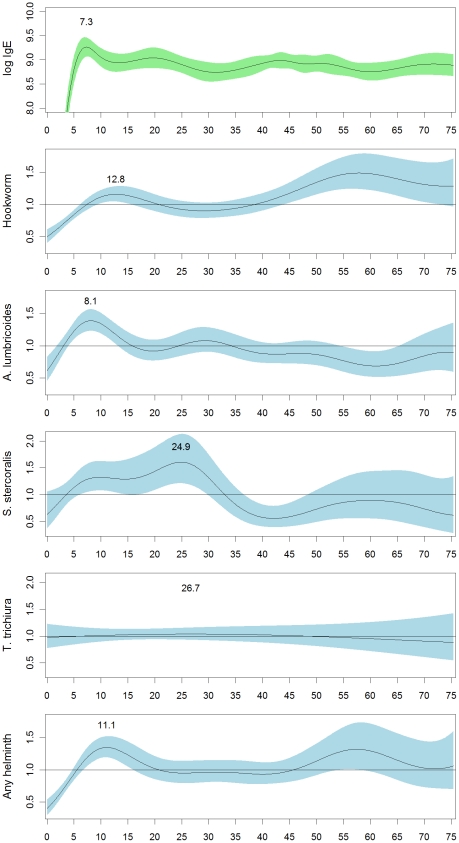
Odds-ratios for Tsimane helminth infection by age relative to the Tsimane population as a whole. Odds-ratios were estimated with generalized additive models with a binomial logit link function. Shading shows the 95% confidence interval for the odds-ratio. All age functions were significant at p<0.001, except the age function for *T. trichiura*, which was non-significant.

Overall, 57% of Tsimane participants were infected with at least one helminth species, with hookworm (45.3%) and *A. lumbricoides* (19.88%) the most prevalent, and *S. stercoralis* (5.6%) and *T. trichiura* (3.2%) less common (Table 4). In order to compare the IgE age-pattern with helminth infection patterns, we examined likelihood of helminth infection by age in the Tsimane sample using logistic GAM models (Figure 4). By sex the only significant difference was in *A. lumbricoides* infection, with women being more likely to be infected (22% vs. 18%, χ^2^ = 15.5, p<.001). By age, the odds-ratio of hookworm infection is highest in adults over age 45, but also has a small peak at age 12.8. In contrast, the odds-ratio for infection with *A. lumbricoides* peaks sharply at age 8.1 and then declines, mirroring the IgE age-pattern more closely. The odds-ratio for infection with *S. stercoralis* peaks somewhat later, around age 24.9. The odds of *T. trichiura* infection is essentially flat with respect to age, reflecting the low prevalence of *T. trichiura*. Overall, the odds-ratio for having any type of helminth infection peaks at age 11.1 and then declines until age 45, at which point it increases again. Odds-ratios closely mirror actual prevalences by age group (Table 4). For infected individuals we also examined whether egg/larva burden showed age-patterning. The only significant age-pattern was a slight decline in hookworm burden with age up to about age thirteen (not shown). Other egg/larva burdens did not show age-patterning independent of changes in detection prevalence.

**Table 4:** Tsimane helminth prevalences by age group.

Age Group	n	Hookworm	*Ascaris* *lumbricoides*	*Strongyloides stercoralis*	*Trichuris trichiura*
0–9	1715	41.3%	22.4%	5.9%	3.1%
10–20	838	48.2%	20.9%	6.7%	3.7%
20–30	598	42.5%	20.1%	8.4%	3.3%
30–40	577	44.4%	20.3%	4.0%	3.3%
40–49	607	49.6%	16.8%	3.6%	3.3%
50–59	342	54.1%	16.4%	4.4%	2.9%
60+	375	53.1%	16.3%	4.3%	2.4%
**Total**	**5,105**	**45.4%**	**19.9%**	**5.6%**	**3.2%**

### Association between Helminth Infection and IgE Levels

**Figure 5:**
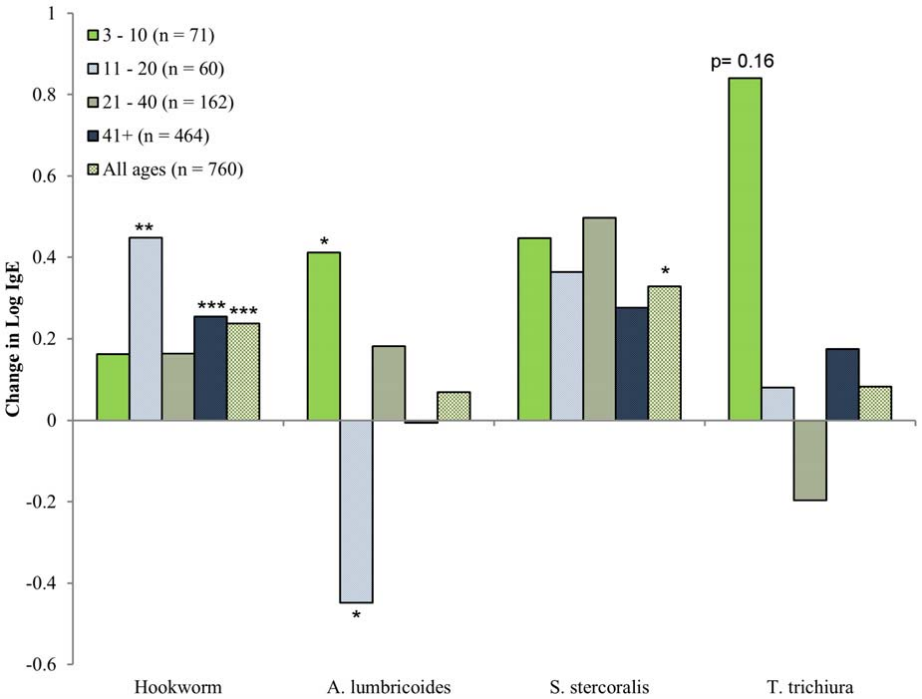
Association between helminth infection and IgE levels by Tsimane age group. Y-values are the regression coefficients from linear models with lnIgE as the dependent variable and infection status for all four parasites entered simultaneously, to control for coinfection status. Models were run separately for each age group indicated. Parameter significance: * p≤0.05, ** p≤0.01, *** p≤0.001.

We examined the association between helminth infection and IgE levels in our Tsimane sample using regression models to control for co-infection status, and with the sample divided by age group (Figure 5). Hookworm infection was significantly associated with higher IgE levels in 11–20 year-olds (β = 0.44, t = 2.85, p<0.01), individuals over forty (β = 0.25, t = 3.35, p<0.01), and in the overall sample (β = 0.23, t = 4.08, p<0.01). *A. lumbricoides* infection was significantly associated with higher IgE levels in individuals ≤10 years-old (β = 0.41, t = 1.96, p = 0.05), but with significantly lower IgE levels in 11–20 year-olds (β = −0.45, t = −2.45, p = 0.02). Although non-significant, *T. trichiura* infection showed a pattern similar to *A. lumbricoides* in those 10 and younger (β = 0.84, t = 1.41, p = 0.16). *S. stercoralis* was positively associated with IgE levels only considering the overall sample (β = 0.32, t = 2.24, p = 0.03).

From the total sample, 459 individuals had IgE levels and full Percoll egg/larva counts. Of these, 195 were positive for hookworm, 89 for *A. lumbricoides*, 18 for *S. stercoralis*, and 16 for *T. trichiura*. Examining infected individuals only, egg/larva counts were not significantly correlated with lnIgE, either in the overall sample or with the sample dived by age.

## Discussion

We report on the age patterning of IgE in three populations: U.S. residents, Ecuadorian Shuar, and Bolivian Tsimane. The highest known IgE levels are found among lowland South American populations [18]. Tsimane IgE levels fit this pattern and resemble the levels of other South American groups with low levels of market integration (e.g., [14], [57], [58]). Tsimane levels are significantly higher than typical values in the United States, even for individuals reporting high levels of allergic symptoms (based on NHANES data, analysis not shown). In contrast, despite inhabiting a similar neotropical environment, Shuar display lower IgE, resembling other South Americans living in rural areas [12], [13], [59]. However, Shuar IgE was also significantly higher than NHANES values.

Although many studies have reported elevated IgE levels in populations infected with parasites such as helminths and malaria, very few have carefully characterized the age-patterning of IgE, and none that we are aware of has tested for a peak shift. A number of studies have noted that IgE is very low at birth, but increases rapidly in the first five years of life [23], [24], [52], [60]. However, most of these studies have been conducted in North America or Europe, and most report that IgE is relatively stable after age five or six without characterizing the degree of stability. One of the few studies to report detailed age profiles found an initial increase to age nine, a slight decrease, and then a second peak at age fifteen in Croatian children [61]. The shape of the increase, with an initial peak and then a second peak, is remarkably similar to the age profiles seen in this study for the NHANES and Shuar sample. Our results suggest that IgE does reach an initial plateau between ages three and five, but continues to increase slowly before reaching higher peaks at age seventeen in the U.S, age ten in the Shuar, and age seven in the Tsimane. It is important to note that although we report the peaks for simplicity, the overall shape of the pattern is more important than the peak itself. This includes a faster rate of increase at an earlier age, an earlier peak, and an earlier decline to adult levels.

The age-patterns we report in this study are consistent with mathematical models for what is known as the peak shift [1], [2], [62]. The peak shift model predicts that immunity will develop earlier in populations with higher exposure and transmission rates and subsequently decline earlier as cohorts acquire partial immunity. The peak shift hypothesis was formulated with regard to helminth infections. Typically, helminth infections peak just before or during adolescence [1], [4], [5], [22]. Although data on helminth infections was only available for one of our three populations, we suggest that the IgE levels and peak ages reported in this study for Shuar and Tsimane are likely the consequence of high helminths loads since helminths are a primary cause of elevated IgE in rural populations. The IgE patterns reported also match expectations from helminth infections. The lack of helminth data for the NHANES participants may also not be much of a limitation, as what studies exist support the assumption that helminths among US residents are likely to be much less prevalent than among either Shuar or Tsimane. There are few recent estimates, but in 1972 Warren estimated that 4.0 million Americans were infected with *A. lumbricoides*, 2.2 million with *T. trichiuris*, 0.7 million with hookworm, and 0.4 million with *S. stercoralis* [63]. Given the US population in 1972, these are prevalences of 1.9%, 1.0%, 0.3%, and 0.2% respectively. Hotez revises Warren's estimate for *S. stercoralis* to a current estimate of 68–100,000 or 0.05% of the 2008 population [64]. Similarly, of 216,275 stool samples sent to state laboratories in 1987, only 0.8% were positive for *A. lumbricoides*, 1.2% for *T. trichiuris*, 1.5% for hookworm, and 0.4% for *S. stercoralis* [65]. A similar study examined 2,896 samples sent to state laboratories in 2000 and found that 0.4% were positive for *A. lumbricoides* [66]. These estimates are clearly much lower than the prevalences we report for Tsimane and the prevalences reported for other ethnic groups living near the Shuar, enough so that the exact prevalence is not critical for interpreting our findings.

Due to T_H_2 biasing, total IgE may be a better index of total helminth load than specific IgE levels. However, the lack of parasite-specific IgE in these data sets is also a limitation in that we cannot state how much parasite-specific IgE contributes to total levels. It may be that Shuar and Tsimane differ less in the total helminth prevalences than they do in prevalences of particular helminth species. Using helminth infection data for the Tsimane we were able to examine associations between helminth species and total IgE. We found that the overall age-pattern for IgE in the Tsimane resembled the age-pattern for *A. lumbricoides* infection. *A. lumbricoides* infection was associated with higher IgE levels in children age 3–10, but with lower IgE levels in 11–20 year-olds. These data suggest that this species may contribute more to the age-pattern of IgE than others. Hookworm and *Strongyloides* infection were also associated with higher IgE in the overall sample, but showed less age-pattern in association. Future studies will need to investigate this in more detail by examining specific-IgE and extending into other populations.

The association between IgE and *A. lumbricoides* is consistent with other studies showing that total IgE is correlated with specific IgE to *A. lumbricoides* [14]. The positive association between *A. lumbricoides* and IgE in participants under age ten and the negative association after age ten may also suggest that IgE conveys partial immunity to *A. lumbricoides* [67], [68]. Other studies that [69] have failed to find increased resistance with higher IgE may have not taken this age-pattern into account.

Other parasites, such as *Plasmodium falciparum*, also raise total IgE levels [70]. However, malaria is unlikely to be an important factor for the populations studied in this paper. Although malaria is present in parts of Shuar territory, it is not present in the villages where the data for this paper were collected, and very few individuals in the area report having had it. Malaria also appears to be absent from the Tsimane territories, with no Tsimane reporting malaria in extensive health interviews.

Finally, in all three populations IgE levels were higher in males. Although noted in many studies (e.g., [23]), the reason for this sex difference is not entirely clear. The only significant sex difference in helminth infections was in *A. lumbricoides*, with slightly more women being infected. Due to the higher IgE in males, it is tempting to hypothesize that this is due to increased resistance in males. However at present this is merely supposition. It is just as likely that Tsimane women are infected more frequently because they spend more time in direct contact with children, who themselves have the greatest number of *A. lumbricoides* infections.

In addition to its importance for theoretical models describing the epidemiology of infections, an understanding of the age patterning of IgE may have public health implications. In populations with higher parasite transmission rates, exposure triggers an elevation of IgE at earlier ages. More rapid and heavy investment in earlier immunocompetence may be favored with high exposure, even at the expense of other investments, such as growth. In Shuar children high IgE levels are associated with increased stunting [18]. It seems plausible that insults to growth may be most pronounced in populations in which peak infection rates occur during critical growth periods, such as early adolescence. Additionally, the timing of infection may affect the development of immune function in other ways, for example by affecting the T_H_1/T_H_2 balance, with consequences for the later development of allergy [71]. Although these hypotheses remain to be tested, they suggest that interventions might be developed with the specific goal of shifting infection peaks toward less critical ages.
